# Endophytic upper tract urothelial carcinoma in a solitary kidney treated by cryotherapy: an unorthodox case for successful management

**DOI:** 10.1186/s12894-023-01279-6

**Published:** 2023-06-27

**Authors:** Ahmad Abdelaziz, Mark Sultan, Muhammed A Hammad, Juan Ramon Martinez, Maria Yacoub, Ramy F Youssef

**Affiliations:** Department of Urology, University of California: Irvine, 3800 Chapman Ave, Suite 7200, Orange, CA 92868 USA

**Keywords:** Upper tract urothelial carcinoma, UTUC, Cryoablation, Nephron sparing surgery

## Abstract

**Background:**

Nephroureterectomy remains the gold standard treatment for upper tract urothelial carcinoma (UTUC). Considering the high risk of developing renal function impairment after surgery, the rationale for nephron sparing approaches in treatment of UTUC has been raised. In this case, renal cryoablation was able to achieve successful oncologic control while preserving renal function during 5 years of follow up without intraoperative or post operative complications.

**Case presentation:**

A 79 year old male presents after three months of macroscopic hematuria. Imaging revealed a 3.6 × 3.1 × 2.7 cm endophytic mass in the interpolar region of the left kidney and an atrophic right kidney. After weighing the lesion’s location with the patient’s of complex medical history, he was counselled to undergo a minimally invasive percutaneous cryoablation as treatment for his solitary renal mass. A diagnostic dilemma was encountered as imaging suggested a diagnosis of renal cell carcinoma. However, the pre-ablation biopsy established an alternative diagnosis, revealing UTUC. Percutaneous cryoablation became an unorthodox treatment modality for the endophytic component of his UTUC followed by retrograde ureteroscopic laser fulguration. The patient was followed in 3 months, 6 months, then annually with cross sectional imaging by MRI, cystoscopy, urine cytology and renal function testing. After five years of follow-up, the patient did not encountered recurrence of UTUC or deterioration in renal function, thereby maintaining a stable eGFR.

**Conclusion:**

Although evidence for nephron-sparing modalities for UTUC is mounting in recent literature, limited data still exists on cryotherapy as a line of treatment for urothelial carcinoma. We report successful management of a low-grade UTUC using cryoablation with the crucial aid of an initial renal biopsy and long-term follow-up. Our results provide insight into the role of cryoablation as a nephron-sparing approach for UTUC.

## Background

The relative rarity of upper urinary tract urothelial tumors (UTUC) is estimated at 5% of all urothelial tumors, 1–2% of all genitourinary tumors, and 7% of all renal tumors [1]. UTUC predominately originates from the urothelial lining of the renal pelvis [2] with tumor stage and grade both validated as independent predictors of mortality [3]. Nephroureterectomy is a well-established gold standard treatment for upper tract urothelial carcinoma [4], as a multicenter review of over 1363 patients treated with radical nephroureterectomy revealed 37.4% of patients had extra organ disease and 63.7% were found to be high grade on histologic review [5]. The most recent guidelines on UTUC corroborate that about 25% of cases present as localized disease, over 50% will have regionally advanced cancer, and 20% will have distant disease [6]. However, management by radical nephroureterectomy is associated with an increased risk of chronic kidney disease (CKD) [7], which is linked to an increased risk of morbid cardiac events and death [8]. This elevated risk may be mitigated for the cohort of patients presenting with localized disease. In addition, recently published literature reports the case specific mortality of UTUC to be higher within rural communities (26.7%) compared to urban centers (15.7%) [9]. One possible etiology for this dichotomy is the increased utilization of novel cross-sectional imaging techniques which has resulted in the frequent detection of low-grade and early-stage tumors [10]. Therefore, we are motivated to explore more conservative approaches to preserve kidney function in the management of early stage UTUC.

Recent reports have affirmed endoscopic management of UTUC as a reasonable option for patients with low-grade pathology on biopsy, without adversely affecting survival outcomes [11]. Though cryoablation has been widely used for renal cell carcinoma (RCC) [12, 13]; data on its application for UTUC is limited. Herein, we present a case for the successful treatment of UTUC through percutaneous cryoablation.

## Case presentation

**Fig. 1:**
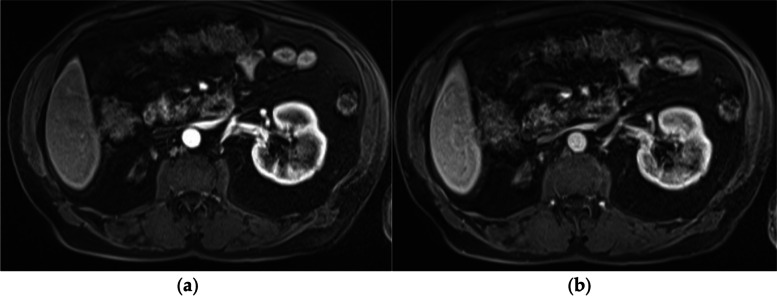
T1 coronal cross-section of the peripherally enhancing lesion with central hypointense signals consistent with both cystic and solid components

A 79-year-old white man presented to clinic with macroscopic hematuria for over 3 months. The patient denied a history of bladder cancer and physical examination revealed no palpable abdominal or renal masses. On multiparametric Magnetic Resonance Imaging (MRI), a lesion measuring 3.6 × 3.1 × 2.7 cm was seen in the interpolar region of the left kidney without involvement of the renal vein or collecting system (Fig. 1). No left sided hydronephrosis or intraabdominal metastasis were appreciated. The right kidney was found to be atrophic. The patient’s medical history was significant for CKD as well as coronary artery disease (CAD) status post coronary artery bypass graft surgery (CABG). Serum creatinine was 1.3 mg./dL, Ca 9.2 mg/dL, albumin 4.2 g/dL, and hemoglobin was 13.7 g/dL. Urine culture was negative. Chest CT revealed no signs of thoracic metastasis.

Initial workup cystoscopy was negative for a bladder mass or bloody efflux from either ureteral orifice, however prostatomegaly was noted. In addition, abdominal MRI images noted endophytic complex cystic mass with solid components making renal cell carcinoma the most likely suspected diagnosis. Considering the patient's comorbidities and the significance of preserving adequate renal function in patients with a solitary kidney, he elected for renal mass biopsy followed by immediate cryoablation as the treatment modality.

The patient was taken to the Interventional Radiology suite for biopsy and cryoablation of the left renal lesion. Under Computerized Tomography (CT) guidance with the patient prone, three 18-gauge core pretreatment biopsies were obtained from the renal mass. Subsequently, three ice-rod probes were distributed across the lesion to maximize treatment margins and two freeze–thaw cycles were carried out. Repeat unenhanced and contrast enhanced CT showed evidence of complete lesion ablation with satisfactory margins. The pathology of the biopsy later confirmed low-grade UTUC. Given this diagnosis was not anticipated, further discussion was warranted regarding the patient’s increased risk of recurrence due to the pathology of his disease. Retrograde ureteroscopy, biopsy, and laser fulguration three months after cryoablation was elected as the next step in management.

**Fig. 2:**
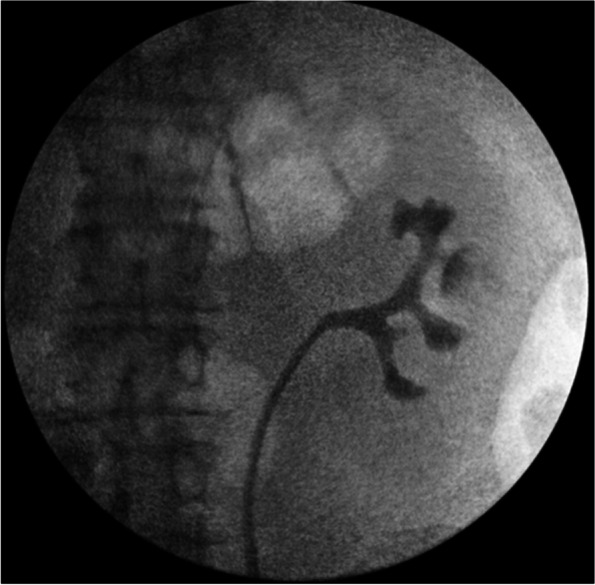
Retrograde Pyelogram of left sided collecting system

Under general anesthesia, full and thorough surveillance cystoscopy was negative for any bladder lesions and left retrograde pyelogram demonstrated no filling defects but an interpolar calyx appeared compressed (Fig. 2). A Wolf fiber optic ureteroscope was utilized for complete pyeloscopy. Yellow-white discoloration with surrounding mucosal edema was visualized in the interpolar calyx consistent with necrotic tissue after cryoablation; however, no obvious papillary fronds of tumor were seen. A Segura four wire basket was deployed to biopsy the superficial necrotic and edematous mucosa followed by BIGopsy biopsy forceps for deep tissue samples. Holmium laser fulguration was applied for bleeding control and obliteration of any potential residual disease. The collected specimens were sent separately. Pathology results from the superficial biopsy demonstrated fragments of non-invasive low-grade papillary urothelial carcinoma. Deeper biopsies revealed cells of uncertain malignant potential in a background of extensive hyaline necrosis and fibrin deposition, corroborating scar hyperplasia and tissue transformation after cryoablation.

**Fig. 3:**
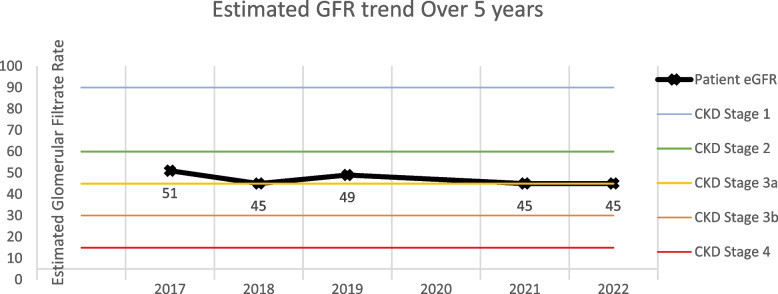
Estimated GFR trend for the patient over 5 years with the lower boundary of each CKD stage highlighted

The patient was monitored over a 5 year period with annual surveillance cystoscopy, bladder cytology, and multiparametric MRI/MRU. To our satisfaction, no visible recurrence of the lesion was observed, and the patient's renal function remained stable (Fig. 3), suggesting the success of this unconventional treatment approach to achieve favorable outcomes. The lack of disease recurrence and preservation of renal function attest to the success of cryoablation in this case.

## Discussion and conclusions

The diagnosis of a renal mass in a solitary kidney presents a challenging clinical scenario given the necessity for conserving renal parenchyma to prevent kidney failure. In such patients, renal compromise may lead to severe consequences as long-term hemodialysis carries a high risk for cardiovascular disease and mortality [14]. To address this challenge, nephron-sparing surgeries were developed as means to adequately maintain kidney function in patients at risk for end stage renal disease, such as those with a solitary kidney, impaired kidney function, bilateral kidney cancer, or those with high likelihood of complications from major surgical procedures [15]. A recent Surveillance Epidemiology and End Results (SEER) database query of 13,075 UTUC patients demonstrated the 5 year case specific mortality for high grade or T3N0M0 disease was 32.0% and 34.5% respectively compared to the 5 year case specific mortality of low grade or T1N0M0 disease at 10.6% and 10.9% respectively [3]. Thus, in select patients with localized, low-grade UTUC, nephron-sparing surgeries have demonstrated satisfactory oncological outcomes while preserving adequate renal function [11]. No prospective randomized trials exist to compare minimally invasive approaches to radical nephroureterectomy as treatment for UTUC. And though the literature regarding cryoablation for UTUC remains limited and inconclusive, this approach in the setting of a solitary kidney limits the overall risk of post-operative renal insufficiency [16]. To reiterate, in the presented five-years of follow-up, the patient exhibited no recurrence of disease or upstaging in CKD.

Cryoablation has been validated as a minimally invasive, yet effective treatment for multiple forms of cancer originating from the eye, brain, head, neck, esophagus, liver, lung, and breast [17–21]. In the field of urology, cryoablation is used to treat RCC and prostate cancer. As a therapy, cryoablation induces cell necrosis through either direct ice formation injury or indirect ischemic effects due to microvascular changes [22]. As the extent of tissue ablation is directly correlated to the number and distance between the probes used, cryoablation remains advantageous for patients with comorbidities as it does not require an invasive surgical technique or a prolonged operative time [23].

The most recent American Urological Association (AUA) guidelines published regarding the management of non-metastatic UTUC advocate for low risk UTUC tumor ablation through a retrograde or anterograde percutaneous approach (Evidence Level: Expert Opinion), however the supporting evidence relies on studies which report the use of holmium, thulium, neodymium (Nd:YAG) or electrocautery as the energy modality for endoscopic UTUC tumor ablation [6]. Thus, as an energy source, the evidence for the use of cryotherapy to manage UTUC remains limited. Furthermore, a recent randomized control trial provided evidence that cryoablation is a safe and efficient therapeutic intervention for non-invasive urothelial carcinoma of the bladder with local disease control in 91% of cases [24]. These findings, in addition to this case report, endorse the potential utility of cryoablation as a treatment option for select patients with non-invasive UTUC.

According to the European Association of Urology’s (EAU) most recently published guidelines panel in 2020, minimally invasive procedures are recommended as primary treatment for UTUC in select cases with a solitary kidney, as disease outcomes are not vastly different compared to nephroureterectomy [25]. The 5-year recurrence rate for UTUC treated with ureteroscopic resection has been reported to be between 2–9% [26]. Tumor grade, multifocality, and a history of bladder cancer have all been reported as predictors of UTUC recurrence [27]. The presented case possessed none of these risk factors. Thus, the nuance of patient selection must again be stressed as this case presented with an approximate 3.5 cm mass on imaging. A study published by Cho et al. reported approximately 80% of upper tract tumors presenting with a diameter > 1.5 cm were found to harbor invasive disease, however 63% of patients with no evidence of hydronephrosis were ultimately diagnosed with pathological T1 or less [28]. Therefore, the lack of obstruction in the setting of an endophytic lesion amenable to endoscopic ablation, as in this case, may portend a more favorable outcome, especially considering low grade UTUC is associated with low rates of metastatic progression [6]. Thus, the value of favorable characteristics such as possessing a unifocal lesion without obstruction, without lymphadenopathy, and without lower tract involvement highlights the necessity of appropriate staging and risk stratification prior to intervention with curative intent.

For this case, a multi-modal approach was essential for the accurate diagnosis of UTUC. CT Urography has been identified as the gold standard imaging for diagnoses due to its high level of accuracy with sensitivity levels reported at 98–99% [29]. The archetypal appearance of UTUC on CT imaging displays an intraluminal enhancing mass with a pedunculated base and a signal intensity of 40–50 HU on the delay phase scans [30]. However, the unusual appearance of this solitary endophytic mass complicated the initial diagnosis supporting renal cell carcinoma as the likely etiology. Retrograde pyelography can also be useful in diagnosis although its low sensitivity of 25% remains a limiting factor [31]. Urine Cytology is also low in sensitivity, with a reported positive rate of only 20–28% for patients presenting with low-grade lesions [31].

The diagnosis of this case posed a substantial challenge due to the patient's renal impairment. Multiparametric MRI was utilized for our diagnosis however select studies have reported a sensitivity of 75% for MRI to accurately diagnose UTUC for lesions less than 2 cm in diameter [32]. However given this patient possessed existing chronic kidney disease as well as a history of atherosclerotic disease, both of which are risk factors for contrast induced acute kidney injury [33], the clinical judgement was to withhold iodinated contrast to limit the potential for contrast induced nephropathy*.* This highlights the crucial role of renal mass biopsy (RMB) in ensuring an accurate diagnosis as imaging independently has a variable sensitivity for predicting histology [34]. No visual evidence of invasion within the deep biopsies of the renal pelvis during the patient’s subsequent endoscopic retrograde biopsy was a valuable result. However, this raises a question regarding whether the prior cryotherapy was successful in eliminating all malignant cells or if obliteration by cryoablation made visual identification of residual tumor on the mucosa inconclusive.

Recently, RMB has become a useful addition to the diagnostic armamentarium for renal tumors supported by data proving its safety and accuracy [35]. Thus, percutaneous intervention for a renal mass without proper biopsy ought be deemed an inadequate diagnostic process, as knowing the oncologic nature of the tissue is key to ensuring an effective treatment. Therefore, the AUA recommends obtaining a tissue diagnosis before cryoablation, as the procedure destroys the cellular architecture, making future pathological diagnosis improbable [36]. In addition, most patients with a renal mass are likely to agree to an initial RMB followed by a discussion of management based on biopsy results. However, some patients would elect to have a single interventional radiology procedure to gain both a renal mass biopsy, and a cryoablation in one session, therefore minimizing procedural and anesthetic risks.

This case report showcases the successful use of cryoablation for the treatment of low grade UTUC. We believe this treatment modality holds potential as a useful addition to the current therapeutic armamentarium. Given conservative management options are valid for low risk UTUC, this case report highlights cryotherapy as a potential ablative energy for treatment of UTUC. Thus further investigation regarding its safety and efficacy are warranted. This will aid in determining its place in the management of this disease and provide a clearer understanding of its potential benefits and limitations. However, the limited scope of the currently available data does not allow for definitive conclusions to be drawn at this time.

## Data Availability

All clinical data was extracted from the University of California, Irvine Electronic Medical Record system (EPIC). Image data was extracted from the clinical PACS and are stored in a DICOM standard format. This data is not available to the public under the Health Insurance Portability and Accountability Act (HIPPA). No datasets were generated or analyzed during the current study. The clinical data is however available from the authors upon reasonable request and with permission of University of California, Irvine.

## Abbreviations

UTUC: Upper urinary tract urothelial carcinoma
CKD: Chronic kidney disease
RCC: Renal cell carcinoma
MRI: Magnetic resonance imaging
CAD: Coronary artery disease
CABG: Coronary artery bypass graft
CT: Computerized tomography
SEER: Surveillance, epidemiology, and end results
AUA: American urological association
EAU: European association of urology
RMB: Renal mass biopsy
